# Role of lysophosphatidic acid in proliferation and differentiation of intestinal epithelial cells

**DOI:** 10.1371/journal.pone.0215255

**Published:** 2019-04-24

**Authors:** Tasuku Konno, Takenori Kotani, Jajar Setiawan, Yuka Nishigaito, Naoki Sawada, Shinya Imada, Yasuyuki Saito, Yoji Murata, Takashi Matozaki

**Affiliations:** Division of Molecular and Cellular Signaling, Department of Biochemistry and Molecular Biology, Kobe University Graduate School of Medicine, Kobe, Japan; National Cancer Institute, UNITED STATES

## Abstract

Intestinal epithelial cells (IECs) are regenerated continuously from intestinal stem cells (ISCs) near the base of intestinal crypts in order to maintain homeostasis and structural integrity of intestinal epithelium. Epidermal growth factor (EGF) is thought to be important to drive the proliferation and differentiation of IECs from ISCs, it remains unknown whether other growth factors or lipid mediators are also important for such regulation, however. Here we show that lysophosphatidic acid (LPA), instead of EGF, robustly promoted the development of intestinal organoids prepared from the mouse small intestine. Indeed, LPA exhibited the proliferative activity of IECs as well as induction of differentiation of IECs into goblet cells, Paneth cells, and enteroendocrine cells in intestinal organoids. Inhibitors for LPA receptor 1 markedly suppressed the LPA-promoted development of intestinal organoids. LPA also promoted the phosphorylation of extracellular signal-regulated kinase (ERK) 1/2 in intestinal organoids, whereas inhibition of mitogen-activated protein kinase/ERK kinase (MEK) 1/2 significantly suppressed the development of, as well as the proliferative activity and differentiation of, intestinal organoids in response to LPA. Our results thus suggest that LPA is a key factor that drives the proliferation and differentiation of IECs.

## Introduction

In the intestine, intestinal epithelial cells (IECs) are regenerated continuously throughout adulthood from intestinal stem cells (ISCs) at the base of intestinal crypts [1, 2]. ISCs self-renew and generate transient amplifying (TA) cells, which are highly proliferative progenies [1, 2]. The TA cells localize above the stem cell niche, divide rapidly, and differentiate into the various IECs such as absorptive enterocytes, mucin-producing goblet cells, peptide hormone–secreting enteroendocrine cells, and antimicrobial peptide–producing Paneth cells. IECs, except Paneth cells, mature and migrate up the crypt toward the tip of intestinal villi. Paneth cells travel down to the base of intestinal crypts and contribute to the stem cell niche by secreting Wnt ligands such as Wnt3 [2, 3]. Eventually, IECs are expelled from the luminal surface of the intestinal epithelium and renew every 3 to 5 days in mouse and human [1, 2]. Although the continuous turnover of IECs is tightly regulated in order to maintain homeostasis of and structural integrity of the intestinal epithelium [1, 2], the detailed molecular mechanisms underlying the regulation of IEC turnover remain poorly understood.

The key factor that drives the proliferative activity of ISCs, as well as of IEC progenitor cells, is likely a major determinant of the turnover rate of mature IECs. The Wnt proteins produced by Paneth cells are thought to play major roles in the maintenance of ISCs [2, 3]. The Wnt signaling pathway is also implicated in the generation of Paneth cells as well as in positive regulation of TA cell proliferation [1, 4]. Notch, through the binding of its ligand Delta, is also thought to be crucial for the maintenance of ISCs, and it controls the balance of absorptive and secretory lineages [5, 6]. By contrast, epidermal growth factor (EGF) is thought to promote the proliferation of TA cells and IECs through activation of the Ras-ERK (extracellular signal–regulated kinase) signaling pathway [2, 7]. However, Ras was also thought to promote the differentiation of both goblet cells and absorptive enterocytes from progenitor cells by counteracting the Wnt signaling pathway [8, 9]. Deletion of Lrig1, a negative regulator of EGF receptor (EGFR) family, causes the crypts expansion and the increased number of ISCs *in vivo* [10], suggesting the importance of EGF for ISC proliferation. It remains unknown whether other growth factors or lipid mediators are also important for the proliferation and differentiation of IECs from ISCs, however. We previously demonstrated that short-chain fatty acids as bacterial fermentation products promoted the proliferation of IECs without EGF [11]. This result indicated the importance of intestinal bacteria for IEC turnover. Like this finding, identification of factors that regulate the proliferation and differentiation of IECs is necessary to understand the detailed molecular mechanisms of IEC turnover. In addition, identification of these factors might promote understanding the intestinal homeostasis and intestinal diseases.

The intestinal organoid is a model of three-dimensional “mini-guts” with crypt-villus domains that contain all the mature IECs [7]. Indeed, EGF is an essential component in the standard culture medium for development of intestinal organoids [7]. Thus, we have here attempted to find another key factor, other than EGF, that promotes the proliferation and differentiation of IECs from ISCs by the use of intestinal organoids.

## Materials and methods

### Ethics statement

This study was approved by the Institutional Animal Care and Use Committee of Kobe University (Permit Number: P150109-R1, P170707), and animal experiments were performed according to Animal Experimentation Regulations of Kobe University. All efforts were made to minimize suffering.

### Mice

C57BL/6J male mice were obtained from CLEA Japan (Tokyo, Japan). These mice were maintained at the Institute for Experimental Animals at Kobe University Graduate School of Medicine under specific pathogen–free conditions.

### Antibodies and reagents

Rabbit polyclonal antibodies (pAbs) to Ki67 (#AM11168PU-S) were obtained from Acris (Herford, Germany). Rabbit pAbs to ERK1/2 (#v1141) were obtained from Promega (Madison, WI). Rabbit pAbs to phosphorylated ERK1/2 (#9101S) were obtained from Cell Signaling Technology (Beverly, MA). Rabbit pAbs to mucin 2 (Muc2) (#sc-15334) were obtained from Santa Cruz Biotechnology (Santa Cruz, CA). Rabbit pAbs to lysozyme (Lys) (#A0099) were obtained from Dako (Glostrup, Denmark). Rabbit pAbs to chromogranin A (CgA) (#ab15160) were obtained from Abcam (Cambridge, MA). Cy3 or Alexa488–conjugated goat secondary antibodies were obtained from Jackson ImmunoResearch (West Grove, PA) and ThermoFisher (Waltham, MA), respectively. Horseradish peroxidase–conjugated goat secondary antibodies were obtained from Jackson ImmunoResearch. U0126 (#9903) was obtained from Cell Signaling Technology. Ki16425 (#sc-221788) was obtained from Santa Cruz Biotechnology. AM095 (#cs-1118) was obtained from Chemscene (Monmouth Junction, NJ). Recombinant murine EGF (#PMG8041) was obtained from ThermoFisher. Recombinant human hepatocyte growth factor (HGF) was kindly provided by K. Matsumoto. Recombinant murine insulin-like growth factor-1 (IGF-1) (#250–19) and recombinant human Trefoil factor 3 (TFF3) (#300–61) were obtained from Peprotech (Rocky Hill, NJ). Sphingosine-1-phosphate (S1P) (#S9666) and LPA (L-α-lysophosphatidic acid, oleoyl, sodium, #L7260) were obtained from Sigma-Aldrich (St. Louis, MO). 4’, 6-diamidino-2-phenylindole (DAPI) was obtained from Nacalai Tesque (Kyoto, Japan).

### Intestinal organoid culture

Intestinal organoid culture was performed as described previously [7, 8, 11], with slight modifications. In brief, crypts were isolated from the small intestine by incubation for 30 min in PBS containing 2 mM EDTA. The isolated crypts were mixed with Matrigel (BD Biosciences) and transferred to 48-well plates. After polymerization of the Matrigel, advanced Dulbecco’s modified Eagle’s medium-F12 (advanced DMEM/F12) (Invitrogen, Carlsbad, CA), which was supplemented with penicillin-streptomycin (100 U/ml) (Invitrogen), 10 mM HEPES (Invitrogen), 1×GlutaMAX (Invitrogen), 1× N2 (Invitrogen), 1× B27 (Invitrogen), 1.25 mM N-acetylcysteine (Sigma-Aldrich), 10% R-spondin1-Fc-conditioned medium, and Noggin (100 ng/ml) (Peprotech), was overlaid on the gel in each well. The crypts were also treated with or without EGF (50 ng/ml), HGF (200 ng/ml), IGF-1 (100 ng/ml), TFF3 (548 ng/ml), S1P (10 μM), or LPA (4 μM). The indicated concentration of each factor maximally promoted the development of intestinal organoids in our preliminary experiments. In some experiments, the crypts were treated with Ki16425 (50 μM) or AM095 (10 μM). The intestinal organoids were then maintained in an incubator (37°C, 5% CO_2_). Phase-contrast images of intestinal organoids were obtained with an Axiovert 200 microscope (Carl Zeiss, Oberkochen, Germany), and the organoid area was measured by encircling the periphery of each organoid with the use of ImageJ software (NIH). The budding number of each organid was visually counted. The data of organoid area and budding number were obtained from randomly selected 30 organoids per experiment in three separate experiments (A total of 90 organoids was analyzed).

### Immunofluorescence analysis

Intestinal organoids in the Matrigel were fixed with 4% paraformaldehyde for 30 min at room temperature and isolated from the gel with Cell Recovery Solution (BD Biosciences). Isolated organoids were then stained with primary antibodies for 3 h at room temperature and stained with fluorescent dye-labeled secondary antibodies as well as with DAPI for 1 h at room temperature. Images were acquired with a confocal laser scanning microscope (LSM710, Carl Zeiss). Pictures were obtained with Z-stack projections of 20 optical slices covering the entire organoid.

### Reverse transcription and polymerase chain reaction (RT-PCR)

Total RNA was prepared from freshly isolated intestinal crypts with the use of RNeasy Mini Kit (Qiagen, Hilden, Germany), and first-strand cDNA was synthesized from 1 μg of the RNA with the use of a QuantiTect Reverse Transcription Kit (Qiagen). The cDNA fragments of interest were amplified by PCR with the primers 5’-ACACCAGCCTGACAGCTTCT-3’ and 5’-CTGTAGAGGGGTGCCATGTT-3’ for LPA receptor 1 (LPA_1_), 5’-TCACTGGTCAATGCAGTGGT-3’ and 5’-AAGGGTGGAGTCCATCAGTG-3’ for LPA receptor 2 (LPA_2_), 5’-AGGGCTCCCATGAAGCTAAT-3’ and 5’-TTCATGACGGAGTTGAGCAG-3’ for LPA receptor 3 (LPA_3_), 5’-TGCATCAGTGTGGATCGTTT-3’ and 5’-GAAGCCTTCAAAGCAAGTGG-3’ for LPA receptor 4 (LPA_4_), 5’-GCTCCAGTGCCCTGACTATC-3’ and 5’-GGGAAGTGACAGGGTGAAGA-3’ for LPA receptor 5 (LPA_5_), 5’-TGTGCCCTACAACATCAACCT-3’ and 5’-CAAAGCAGCAGTTGGAAACA-3’ for LPA receptor 6 (LPA_6_).

### Immunoblot analysis

Isolated crypts were cultured in the Matrigel with advanced DMEM/F12 supplemented with penicillin-streptomycin (100 U/ml), 10 mM HEPES, and 1×GlutaMAX overnight. Then formed organoids were stimulated with EGF (50 ng/ml) or LPA (4 μM) for 0–60 min and they were harvested from the Matrigel with Cell Recovery Solution. Organoids were then washed with ice-cold PBS and lysed with lysis buffer [20 mM Tris-HCl (pH 7.5), 150 mM NaCl, 2 mM EDTA, 1% Nonidet P-40, 1% sodium deoxycholate, 0.1% sodium dodecyl sulfate, 50 mM NaF] containing 1 mM sodium vanadate and a protease inhibitor cocktail (Nacalai Tesque). The resulting lysates were subjected to immunoblot analysis as previously described [11].

### Statistical analysis

Data are presented as means ± standard errors of the means (SEM). Unpaired, two-tailed Student's *t* tests were used to compare between 2 groups. One-way ANOVA followed by Tukey's tests were used to compare among 3 groups. Results were analyzed using GraphPad Prism software version 6.0 (GraphPad Software). A *P* value of <0.05 was considered statistically significant.

## Results

### Effects of LPA on the development of intestinal organoids

**Fig 1:**
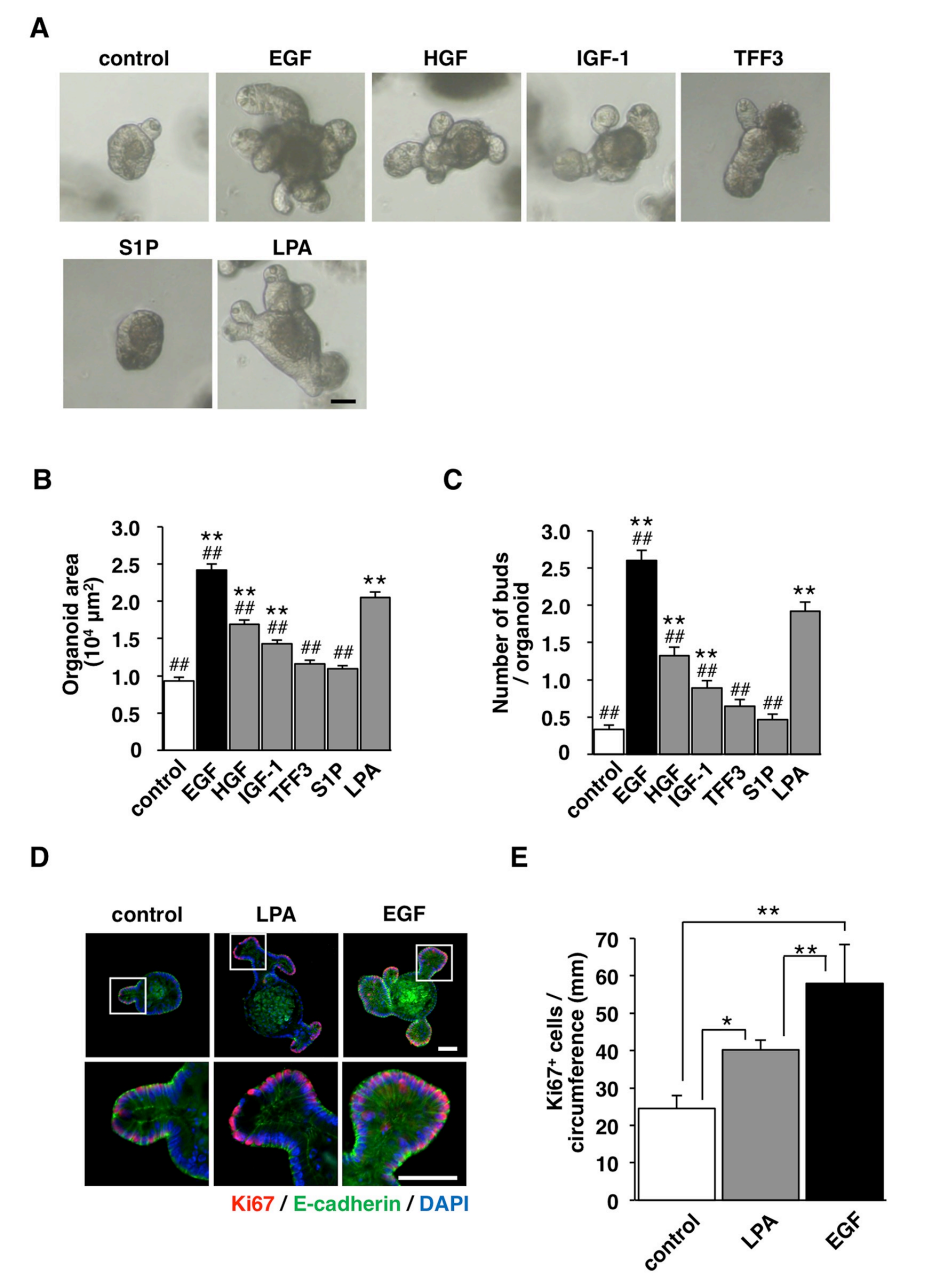
Promotion by LPA of the development of intestinal organoids. (**A**) Isolated crypts were cultured with or without (**control**) various growth factors or lipid mediators indicated for 4 days. Scale bar, 50 μm. (**B**) Areas of intestinal organoids cultured as for (A) were determined. (**C**) The number of buds per organoid cultured as for (A) was determined. Quantitative data in (B) and (C) are means ± SEM for a total of 90 organoids per group in three separate experiments. **, *P* < 0.01 vs control; ##, *P*<0.01 vs LPA (ANOVA and Tukey’s test). (**D**) Intestinal organoids cultured with or without (**control**) LPA or EGF for 4 days were subjected to immunostaining with antibodies to Ki67 (red) and to E-cadherin (green), and to staining of nuclei with DAPI (blue). The boxed areas in the upper panels are shown at higher magnification in the lower panels. Scale bars, 50 μm. (**E**) The number of Ki67-positive (Ki67^+^) cells normalized to the circumference of each organoid was determined. Quantitative data in (E) are means ± SEM for a total of 30 organoids per group in three separate experiments. *, *P* < 0.05; **, *P* < 0.01 (ANOVA and Tukey’s test).

We first examined the effects of various growth factors as well as lipid mediators on the development of intestinal organoids. Isolated crypts from the small intestine were cultured with or without growth factors or lipid mediators in the Matrigel containing R-spondin1 and Noggin (see Materials and Methods). The effect of each growth factor or lipid mediator on the development of intestinal organoids was evaluated by measurements of both organoid size and the number of buds at day 4 of the culture (**Fig 1A–1C**). As previously described by Sato et al. [7], we confirmed that EGF promoted the development of intestinal organoids compared that apparent for the control (no growth factors) (**Fig 1A–1C**). HGF and IGF-1, both of which effects are thought to be mediated by their receptor tyrosine kinase, also promoted the development of intestinal organoids, their effect was much smaller than that of EGF, however (**Fig 1A–1C**). In addition, either TFF3, a growth factor-like peptide secreted partly by goblet cells [12], or S1P, of which effects are mediated by the receptor-coupled to G-protein [13], did not significantly promote the development of intestinal organoids (**Fig 1A–1C**). In contrast, LPA promoted the development of intestinal organoids (**Fig 1A–1C)**; the effect was smaller than that apparent for EGF, but it was significantly larger than other factors tested (**Fig 1A–1C)**. Immunostaining for Ki67, a marker of proliferating cells, showed that LPA increased the number of Ki67-positive cells compared to the control (**Fig 1D and 1E**), whereas the effect was less potent compared with that of EGF. These results suggest that LPA markedly promotes the proliferation of IECs and development of intestinal organoids.

### Effects of LPA on the differentiation of IECs in intestinal organoids

**Fig 2:**
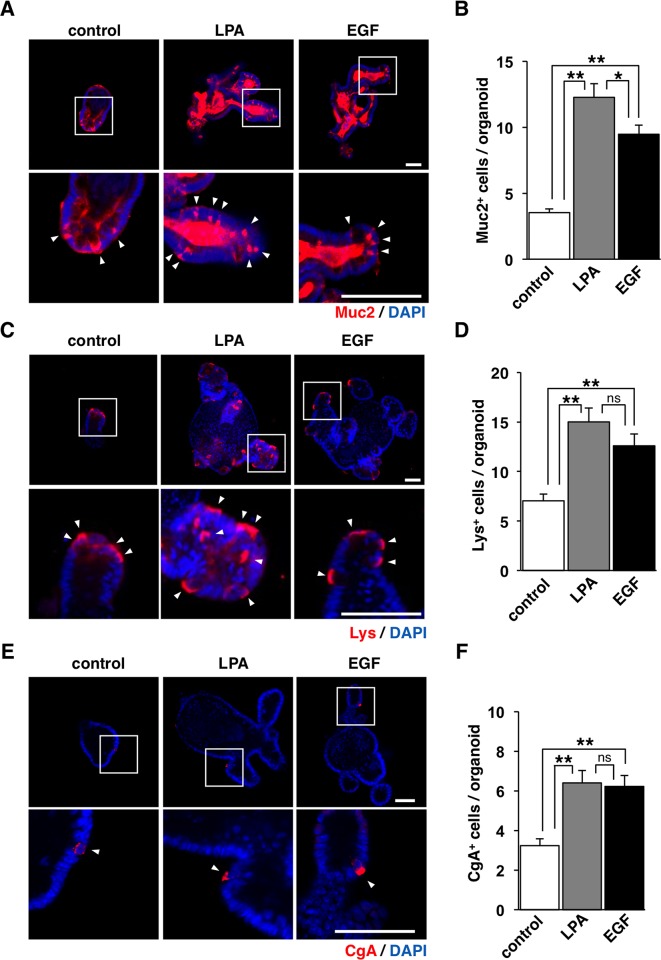
Differentiation of intestinal organoids induced by LPA. (**A**) Intestinal organoids cultured with or without (**control**) LPA or EGF for 4 days were subjected to immunostaining with an antibody to Muc2 (red) and to staining of nuclei with DAPI (blue). (**B**) The number of Muc2-positive (Muc2^+^) cells per organoid in (A) was determined. (**C**) Intestinal organoids cultured as for (A) were subjected to immunostaining with an antibody to Lys (red) and to staining of nuclei with DAPI (blue). (**D**) The number of Lys-positive (Lys^+^) cells per organoid in (C) was determined. (**E**) Intestinal organoids cultured as for (A) were subjected to immunostaining with an antibody to CgA (red) and to staining of nuclei with DAPI (blue). (**F**) The number of CgA-positive (CgA^+^) cells per organoid in (E) was determined. The boxed areas in the upper panels are shown at higher magnification in the lower panels. Arrowheads indicate Muc2^+^ (A), Lys^+^ (C), or CgA^+^ (E) cells. Scale bars, 50 μm. Quantitative data are means ± SEM for a total of 30 organoids per group in three separate experiments. *, *P* < 0.05; **, *P* < 0.01; ns, not significant (ANOVA and Tukey’s test).

We next investigated the effects of LPA on the differentiation of IECs in intestinal organoids. Intestinal organoids include various types of differentiated IECs such as absorptive enterocytes, goblet cells, Paneth cells, and enteroendocrine cells [7]. Mucin 2 (Muc2), lysozyme (Lys), and chromogranin A (CgA) are known as markers for goblet cells, Paneth cells, and enteroendocrine cells, respectively [7]. LPA increased the number of Muc2-positive goblet cells compared with the control and such effect was significantly greater than that apparent for EGF (**Fig 2A and 2B**). In addition, LPA increased the number of Lys-positive Paneth cells (**Fig 2C and 2D**) and CgA-positive enteroendocrine cells (**Fig 2E and 2F**) compared with the control; the extents of such effects were similar to that apparent for EGF (**Fig 2C–2F**). These results thus suggest that LPA markedly promotes the differentiation of IECs.

### Importance of LPA receptor 1 (LPA_1_) for the LPA-promoted development of intestinal organoids

**Fig 3:**
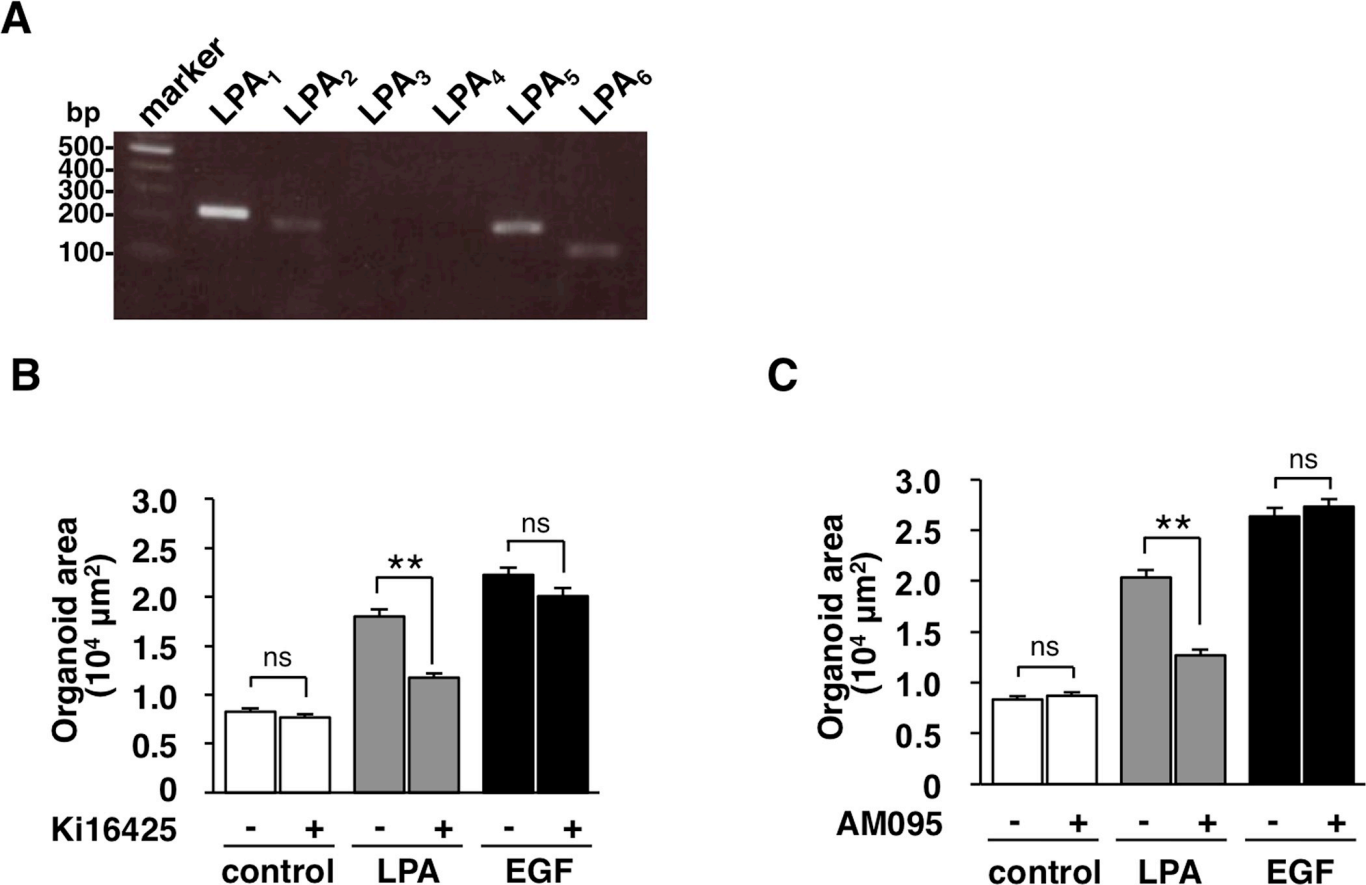
Importance of LPA_1_ for the promotion of the development of intestinal organoids by LPA. (**A**) Expressions of LPA receptors in intestinal crypts were tested by RT-PCR. (**B**) Intestinal organoids cultured with or without (**control**) LPA or EGF were treated with (**+**) or without (**-**) Ki16425 for 4 days. Areas of intestinal organoids were then determined. (**C**) Intestinal organoids cultured with or without (**control**) LPA or EGF were treated with (**+**) or without (**-**) AM095 for 4 days. Areas of intestinal organoids were then determined. Quantitative data are means ± SEM for a total of 90 organoids per group in three separate experiments. **, *P* < 0.01; ns, not significant (ANOVA and Tukey’s test).

Given that six subtypes of receptors for LPA have been described so far [14], we next investigated which subtypes of LPA receptors were expressed in the isolated intestinal crypts. By the use of RT-PCR, we detected the expression of mRNAs for LPA receptor subtypes, LPA_1_, LPA_2_, LPA_5_, and LPA_6_, in the intestinal crypts (**Fig 3A**). Previous study has also reported that the mRNA expression for LPA_1_ is the most abundant among LPA receptor subtypes in the mouse small intestine [15]. Ki16425, a selective antagonist for LPA_1_, LPA_2_, and LPA_3_ [16], markedly suppressed the LPA-promoted development of intestinal organoids (**Fig 3B**). In addition, AM095, a potent antagonist against LPA_1_ [17], also suppressed the LPA-promoted development of intestinal organoids (**Fig 3C**), whereas both antagonists did not suppress either the control or the EGF-promoted organoid development (**Fig 3B and 3C**). These results suggest that LPA_1_ is important for the LPA-promoted development of intestinal organoids.

### Importance of MEK (mitogen-activated protein kinase/ERK kinase) -ERK signaling pathway for the LPA-promoted development of intestinal organoids

**Fig 4:**
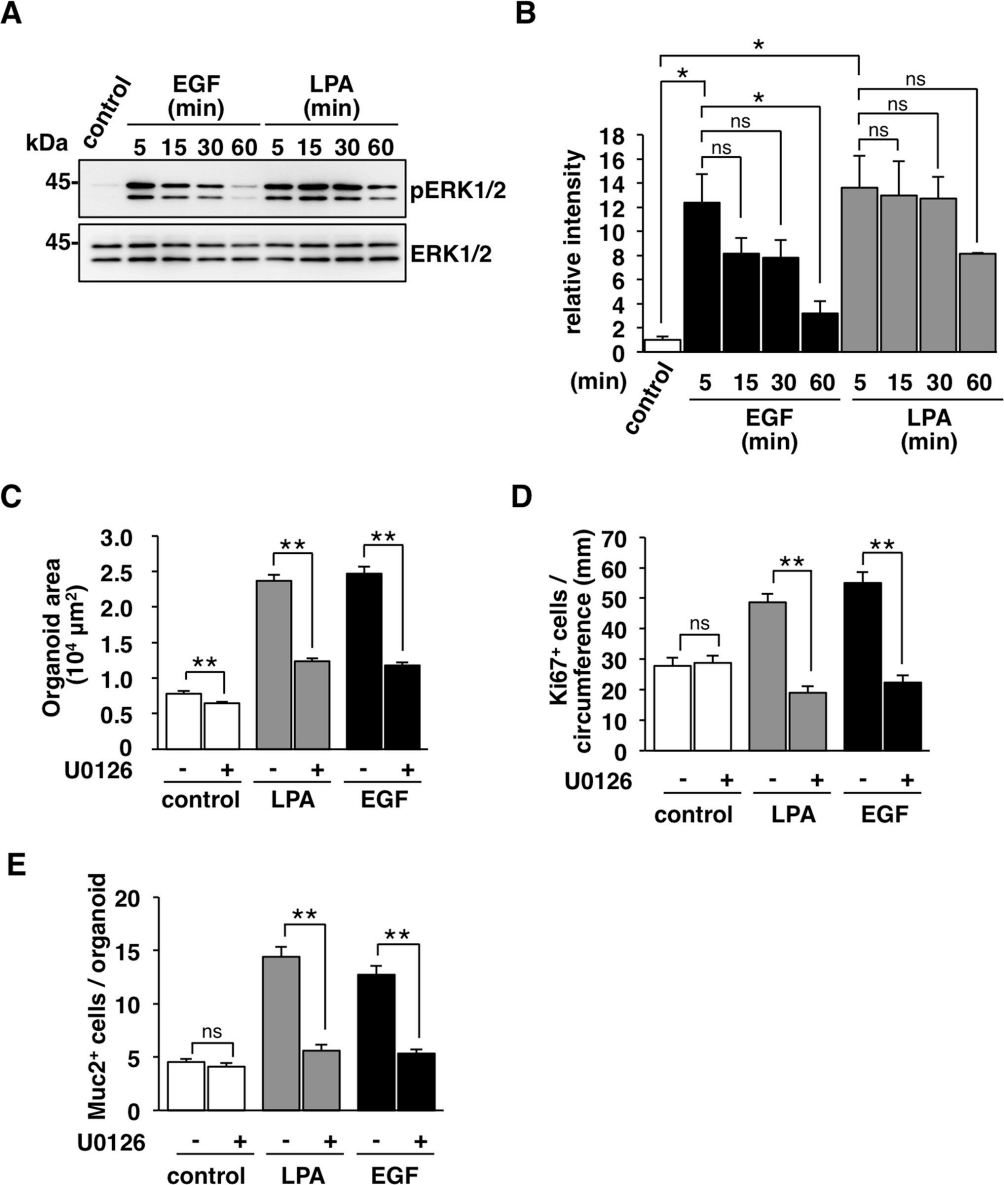
Importance of MEK-ERK signaling pathway for the promotion of the development, proliferative activity, and differentiation of intestinal organoids by LPA. (**A**) Intestinal organoids were stimulated with or without (**control**) LPA or EGF for indicated timepoints, after which they were subjected to immunoblot analysis with antibodies to phosphorylated ERK1/2 (pERK1/2) or ERK1/2. (**B**) The band intensity in (A) for pERK1/2 was normalized by that for ERK1/2 and expressed relative to the normalized value for the control. (**C**) Intestinal organoids cultured with or without (**control**) LPA or EGF were treated with (**+**) or without (**-**) U0126 for 4 days. Organoid area was then determined. Quantitative data are means ± SEM for a total of 90 organoids per group in three separate experiments. (**D, E**) The numbers of Ki67- (D) or Muc2- (E) positive cells in intestinal organoids cultured as for (C) were determined. Quantitative data are means ± SEM for a total of 30 organoids per group in three separate experiments. **, *P* < 0.01; ns, not significant (ANOVA and Tukey’s test).

LPA_1_ as well as EGFR are thought to promote activation of the Ras-MEK-ERK signaling pathway [14], we next examined whether this signaling pathway is important for the effect of LPA on the development of intestinal organoids. We found that both LPA and EGF induced the marked phosphorylation of ERK1/2; the effect of LPA was longer than that of EGF (**Fig 4A and 4B**). In addition, U0126, an inhibitor of MEK1/2 [18], significantly attenuated the development of the LPA-, as well as EGF-, promoted development of organoids (**Fig 4C**). Moreover, U0126 markedly suppressed the LPA-induced increase of Ki67-positive cells (**Fig 4D**) or that of Muc2-positive goblet cells, with the similar inhibition by U0126 for the effect of EGF (**Fig 4E**). These results thus suggest that the MEK-ERK signaling pathway is important for the LPA-promoted proliferation and differentiation of IECs in intestinal organoids.

## Discussion

EGF is thought to have a potent effect for proliferation of IECs and development of intestinal organoids. However, we have shown that HGF and IGF-1, both of which effects are also mediated by their receptor tyrosine kinase, promoted the development of intestinal organoids, but their effects were smaller than that of EGF. In contrast, we here for the first time showed that LPA, of which effects are mediated by G protein-coupled receptors, promoted the proliferation of IECs and development of intestinal organoids. The effect of LPA on the organoid development was higher than that of HGF or IGF-1. In addition, LPA increased the numbers of Muc2-positive goblet cells, Lys-positive Paneth cells, and CgA-positive enteroendocrine cells in intestinal organoids. A previous study showed that LPA_1_-deficient (*Lpar1*^-/-^) mice manifested the decreased proliferative activity of IECs in intestinal crypts as well as the impaired migration of these cells along the crypt-villus axis [19]. Consistently, we found that LPA_1_ is important for the LPA-promoted development of intestinal organoids. A previous study also showed that LPA enhanced the proliferation and migration of immortalized mouse colonic epithelial cells through PLC-β1 and PLC-β2 [19]. Here, we described a novel finding that the activation of the MEK-ERK signaling was essential for the LPA-induced proliferation and differentiation of IECs by the use of intestinal organoids. Indeed, we and others recently showed that Ras-ERK signaling pathway is important for proliferation and differentiation of IECs [8, 9, 20]. Interestingly, previous studies showed that LPA had an ability to induce ectodomain shedding of EGFR ligands [21]. Thus, LPA might shed EGFR ligands on IECs and induce the activation of the Ras-MEK-ERK signaling through EGFR in IECs. The study of *Lpar1*^-/-^ mice also showed that LPA_1_ is important for epithelial wound repair after dextran sulfate sodium (DSS)-induced colitis in mice [19]. In addition, administration of LPA is known to reduce the severity of DSS-induced colitis in mice [22]. Given that the intestinal organoid mimics the regeneration of IECs from the intestinal progenitors, our present study further strengthen an idea that LPA is a potent stimulant for regeneration of IECs and likely serves a therapeutic tool for colitis. Further investigation will be required to clarify the action of LPA for the proliferation and differentiation of IECs and its clinical application.

## Supporting information

S1 Table Values used to build graphs. (XLSX)
